# Targeting endogenous DLK1 exerts antitumor effect on hepatocellular carcinoma through initiating cell differentiation

**DOI:** 10.18632/oncotarget.12214

**Published:** 2016-09-23

**Authors:** Chun-Miao Cai, Xu Xiao, Bing-Hao Wu, Bao-Feng Wei, Ze-Guang Han

**Affiliations:** ^1^ Key Laboratory of Systems Biomedicine (Ministry of Education) and Collaborative Innovation Center of Systems Biomedicine of Rui-Jin Hospital, Shanghai Jiao Tong University School of Medicine, Shanghai 200025, China; ^2^ Shanghai-MOST Key Laboratory for Disease and Health Genomics, Chinese National Human Genome Center at Shanghai, Shanghai 201203, China; ^3^ Shanghai Center of Systems Biomedicine, Shanghai Jiao Tong University, Shanghai 200240, China

**Keywords:** DLK1, hepatocellular carcinoma, RNA interference, cell differentiation

## Abstract

Cancer stem cells (CSCs) are responsible for tumor initiation and progression. We previously showed that Delta-like homolog 1 (DLK1) may be a therapeutic target against the CSCs of human hepatocellular carcinoma (HCC). However, the therapeutic efficacy and underlying mechanism remain unclear. Here we demonstrated that knockdown of *DLK1* using a tet-inducible short hairpin RNA (shRNA) system significantly inhibited proliferation, spheroid formation and *in vivo* xenograft tumor growth of human HCC cells. Furthermore, in an orthotopic xenograft mouse model, adenovirus-mediated *DLK1* knockdown could significantly reduce tumor size, as shown by *in vivo* imaging approach. Subsequently, an adenoviral vector harboring mouse *Dlk1* shRNA was applied. The results showed that Dlk1 knockdown also could inhibit tumor progression in a diethylnitrosamine (DEN) induced mouse HCC model. At cellular mechanism, *DLK1* knockdown delayed the cell cycle G1-S transition, along with the decreased expression of cyclin E1 and D1. Significantly, *DLK1* knockdown resulted in the decrease of molecular markers such as *AFP* and *EpCAM* for hepatic progenitor cells, but the increase of *KRT18* and *KRT19* for the differentiated hepatocytes. The collective data indicated that targeting endogenous DLK1 may exert antitumor effect on HCCs possibly through initiating cell differentiation.

## INTRODUCTION

Hepatocellular carcinoma (HCC) is a common cancer and the third leading cause of death from cancer worldwide [1]. Currently, the first-line treatment is surgical resection or liver transplantation, but only a small proportion of patients are qualified for the either [2, 3]. Chemotherapy is the second-line treatment for most HCC patients who are found to be present at advanced stages. However, the overall response rate is unsatisfactory as these conventional agents only kill rapidly dividing cells but not a minority of cells in relative quiescence [4–6], which are called cancer stem cells (CSCs). Therefore, it is urgent to develop new therapeutic agents against the CSCs of HCC.

Significantly, delta-like homolog 1 (DLK1), a non-canonical Notch ligand, is widely expressed during embryonic development [7, 8]. Postnatally, its expression is restricted to few tissues [9–11]. Many studies demonstrate that expression of DLK1 is elevated in a wide range of tumor types, including neuroblastoma, gliomas, breast cancer, colon cancer, pancreatic cancer, small-cell lung carcinoma, leukemia and hepatocellular carcinoma [12–18]. Our previous studies indicated that DLK1 was overexpressed in human HCC specimens and the ectopic DLK1 could promote proliferation of HCC cells [19]. Besides, DLK1^+^ cells manifested CSC-like properties, including chemoresistance, self-renewal ability and xenograft tumorigenesis [20]. Therefore, DLK1 is considered as a potential biomarker and therapeutic target against the CSCs of HCC.

However, the therapeutic efficacy and mechanism of targeting DLK1 in antitumor activities remain unclear. In this study, we evaluated the therapeutic efficacy by animal models, including an orthotopic liver xenograft model derived from human HCC cells, and a chemically-induced mouse HCC model. Our results documented the antitumor effect of targeting endogenous DLK1 on human and mouse HCCs may initiate cell differentiation of CSCs, along with the dysregulation of active and negative cell cycle regulators.

## RESULTS

### Doxycycline (Dox)-triggered *DLK1* knockdown inhibits colony formation and spheroid formation of HCC cells

**Figure 1:**
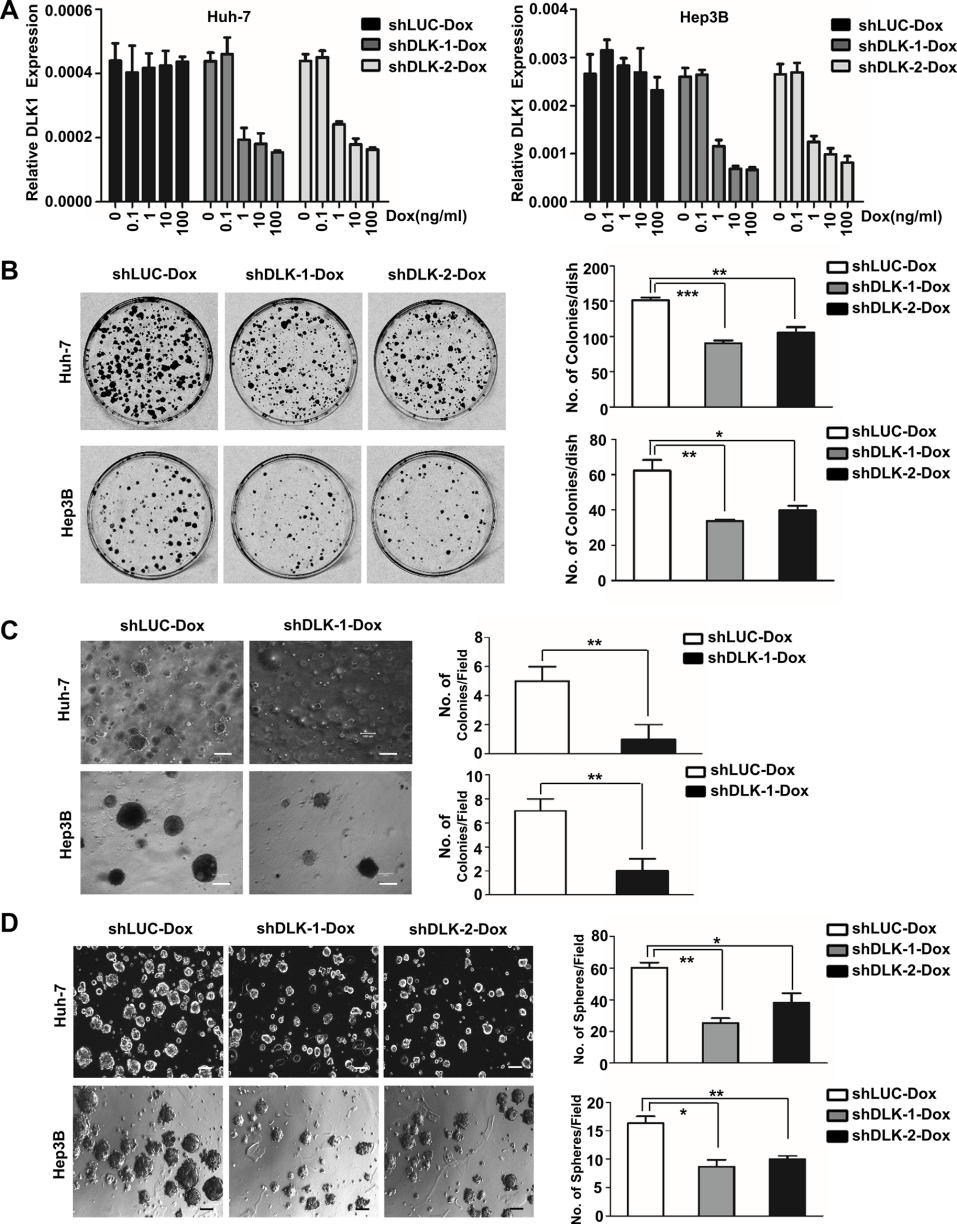
The inducible DLK1 knockdown can inhibit proliferation and colony formation of HCC cells (**A**) Quantitative RT-PCR was used to evaluate the efficacy of the inducible shRNA against endogenous DLK1 through examining *DLK1* mRNA in Huh-7 and Hep3B cells with stably ectopic plasmids as Dox treatment. The Dox-induced DLK1 knockdown may inhibit colony formation (**B**), anchorage-independent growth, as shown by *in vitro* soft-agar assay (**C**), and spheroid colony formation (**D**) derived from Huh-7 and Hep3B cells. Plasmid shLUC encoding shRNA against luciferase was used as control. The representative images are shown and statistical analyses are performed (right). The colony formation data were analyzed by one-way ANOVA. For comparison with control, Student's *t*-test was performed. Data of soft-agar assay were analyzed by unpaired, two-tailed Student's *t*-test was performed for shDLK-1-Dox versus control (*n* = 3, for both two cell lines). The spheroid formation data were also first analyzed for both two cell lines by one-way ANOVA, followed by comparison with control by Student's *t*-test. *p < 0.05; **p < 0.01; ***p < 0.001.

To further validate efficient effect of DLK1 acting as therapeutic target of HCC, two shRNA sequences [21] were inserted into a conditional inducible knockdown system pLKO-tet-on [22], and then stably introduced into three HCC cell lines Huh-7, Hep3B and HepG2 that express *DLK1* (Figures S1A and S1B). As was expected, the endogenous *DLK1* was knocked down by the inducible pLKO-tet-on system triggered by Dox in a dose-dependent manner (Figure 1A and Figure S1C).

Subsequently, we observed the effect of *DLK1* knockdown induced by Dox (10 ng/ml) on colony formation, revealing that the number of colonies formed on agarose plate and in soft agar was significantly reduced, as compared to the controls (Figure 1B and 1C). We also further evaluated the effect of *DLK1* knockdown on spheroid formation of HCC cells due to DLK1 as potential biomarker of tumor stem/progenitor cells of HCCs [20]. The results showed that spheroid formation of Huh-7 and Hep3B cells was significantly inhibited by the inducible *DLK1* knockdown (Figure 1D). Besides, the spheroids formed again in both stable cells by recombinant DLK1 reintroduction, showing larger diameter than those with *DLK1* knockdown, although smaller diameter as compared to the spheroids formed in control stable cells containing *luciferase* shRNA (Figure S1D). These results indicated that the ability of self-renewal of HCC stem/progenitor cells was inhibited by *DLK1* knockdown.

### DLK1 knockdown suppresses growth of *in vivo* xenograft tumors

**Figure 2:**
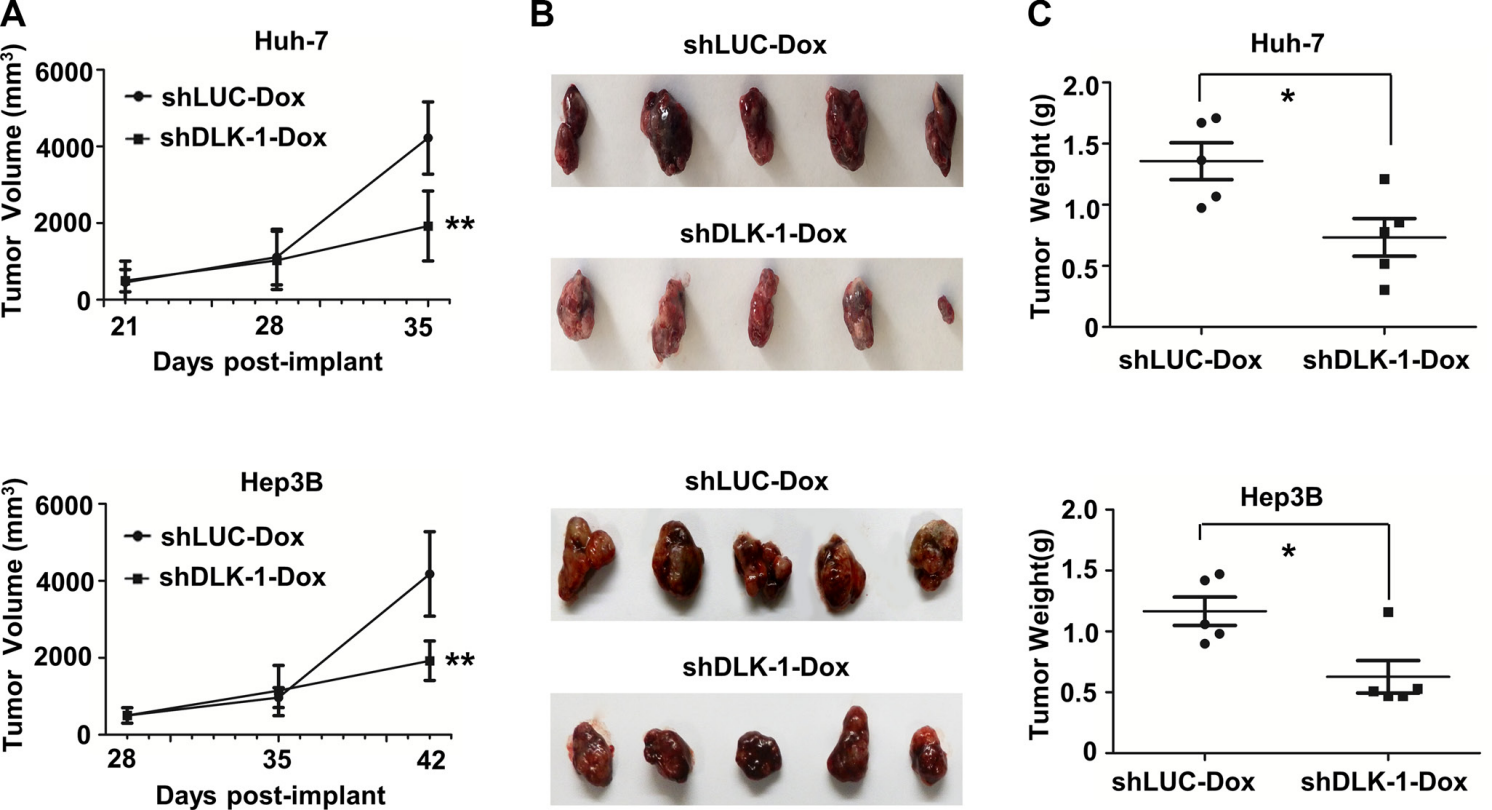
DLK1 knockdown can suppress xenograft tumor growth in nude mice (**A**) The growth curves of xenograft tumors derived from Huh-7 and Hep3B cells with inducible DLK1 knockdown; (**B**) The images of xenograft tumors with inducible DLK1 knockdown; (**C**) Weight of xenograft tumors with inducible *DLK1* knockdown was significantly reduced. In this study, nude mice were given 1 mg/ml of Dox for 15 days as xenograft tumors reached 4 mm in diameter, and shLUC encoding shRNA against ectopic luciferase was used as control. *p < 0.05; **p < 0.01.

To determine whether *DLK1* knockdown can suppress tumor growth when tumors already exist, we inoculated nude mice with Huh-7 and Hep3B stable cells expressing inducible *DLK1* shRNA. When the xenograft tumors reached a volume of 150–200 mm^3^, we employed Dox dissolved in drinking water as 1 mg/ml concentration to trigger the endogenous DLK1 knockdown of the xenograft HCCs. Observed during 21 to 42 days post inoculation, the xenograft HCCs with the inducible *DLK1* knockdown exhibited a significant reduction of tumor volume and weight, as compared to the controls with *luciferase* knockdown (Figures 2A–2C, S2B and S2C). To further confirm the effect of DLK1 knockdown on *in vivo* tumorigenicity, one of the above efficient *DLK1* shRNA sequences was subcloned into the recombinant lentiviral vector, and then stably transfected into Huh-7 and Hep3B HCC cells. As expected, the growth of xenograft tumors derived from both Huh-7 and Hep3B stable cell lines with *DLK1* knockdown was significantly inhibited as compared to that of control with *luciferase* knockdown (Figure S2A). The collective data indicated that targeting endogenous DLK1 may suppress the *in vivo* tumorigenicity of human HCC cells.

### RNAi-mediated DLK1 knockdown reduces tumor growth in an orthotopic xenograft model

**Figure 3:**
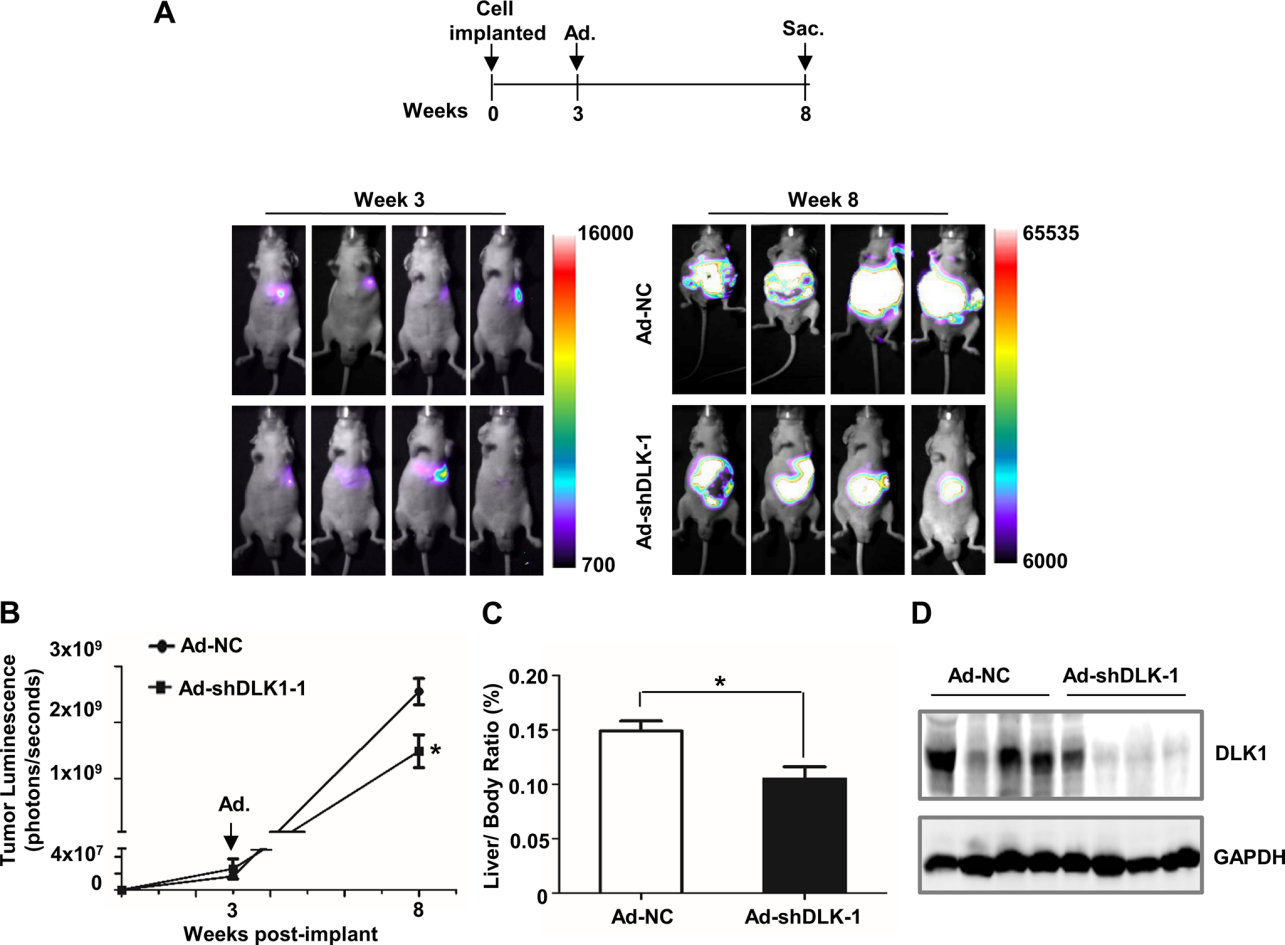
DLK1 knockdown mediated by adenovirus administration inhibits orthotopic xenograft tumor growth in athymic mice (**A**) Scheme and representative pseudocolor images of orthotopic xenograft tumors within nude mice at 3 and 8 weeks after inoculation. 2 × 10^6^ Huh-7 cells expressing luciferase were injected into the left liver lobe of nude mice. Luciferase imaging of these mice was performed once a week until tumor luminescence was observed, and then recombinant adenoviruses were administered at week 3 post cell implantation by tail vein injection. Luminescence imaging of these mice was performed at week 8 after inoculation. (**B**) Tumor luminescence was analyzed. Data represent the mean of 4 mice for each group ± sem (error bars). (**C**) Liver/body weight ratio was determined when these nude mice were sacrificed. (**D**) Western blotting assay was used for evaluating the efficiency of adenovirus-mediated DLK1 knockdown in these orthotopic xenograft tumors. Adenoviral vector containing scrambled shRNA was used as negative control. *p < 0.05.

To further evaluate the therapeutic efficacy of DLK1 knockdown in xenograft tumors of human HCC cells, we constructed the stable luciferase-expressed Huh-7 cell line, and then generated an orthotopic liver xenograft model. Eight mice were orthotopically injected with the established Huh-7 cells, and tumor growth was measured each week by bioluminescent imaging (BLI) until tumor light emission was observed at week 3 (Figure 3A). After that, half of mice were tail-vein injected with adenovirus vector encoding shRNA against *DLK1*,; whilst the remaining as control were injected using scramble vector. After 5 weeks post tail vein injection, luciferase level and activity were assessed by whole-body imaging for tumor light emission. Interestingly, luciferase signal from the xenograft tumors in these mice was significantly attenuated, as compared to that from controls with scramble vector (Figure 3A and 3B). These harvested xenograft tumors also exhibited lower weight and DLK1 expression (Figure 3C and 3D). These data revealed that targeting DLK1 exhibits *in vivo* antitumor activities for HCC cells.

### Adenovirus-mediated DLK1 knockdown suppresses tumor progression in the DEN-induced mouse HCC model

To further evaluate the antitumor efficacy of DLK1 as a therapeutic target, here we employed the DEN-induced mouse HCC model as the experimental subject [23]. Like human HCC, DLK1 was also obviously elevated in the mouse HCC tumors, as shown by immunofluorescence and quantitative RT-PCR detection (Figure S3A and S3B). We then constructed a recombinant adenoviral vector encoding shRNA against mouse *Dlk1*. In addition to knocking down the endogenous *Dlk1* in NIH-3T3 cells (Figure S3C), the recombinant adenoviral vector also efficiently entered mouse livers 10 days after tail vein injection, as documented by GFP expression (Figure S3D).

**Figure 4:**
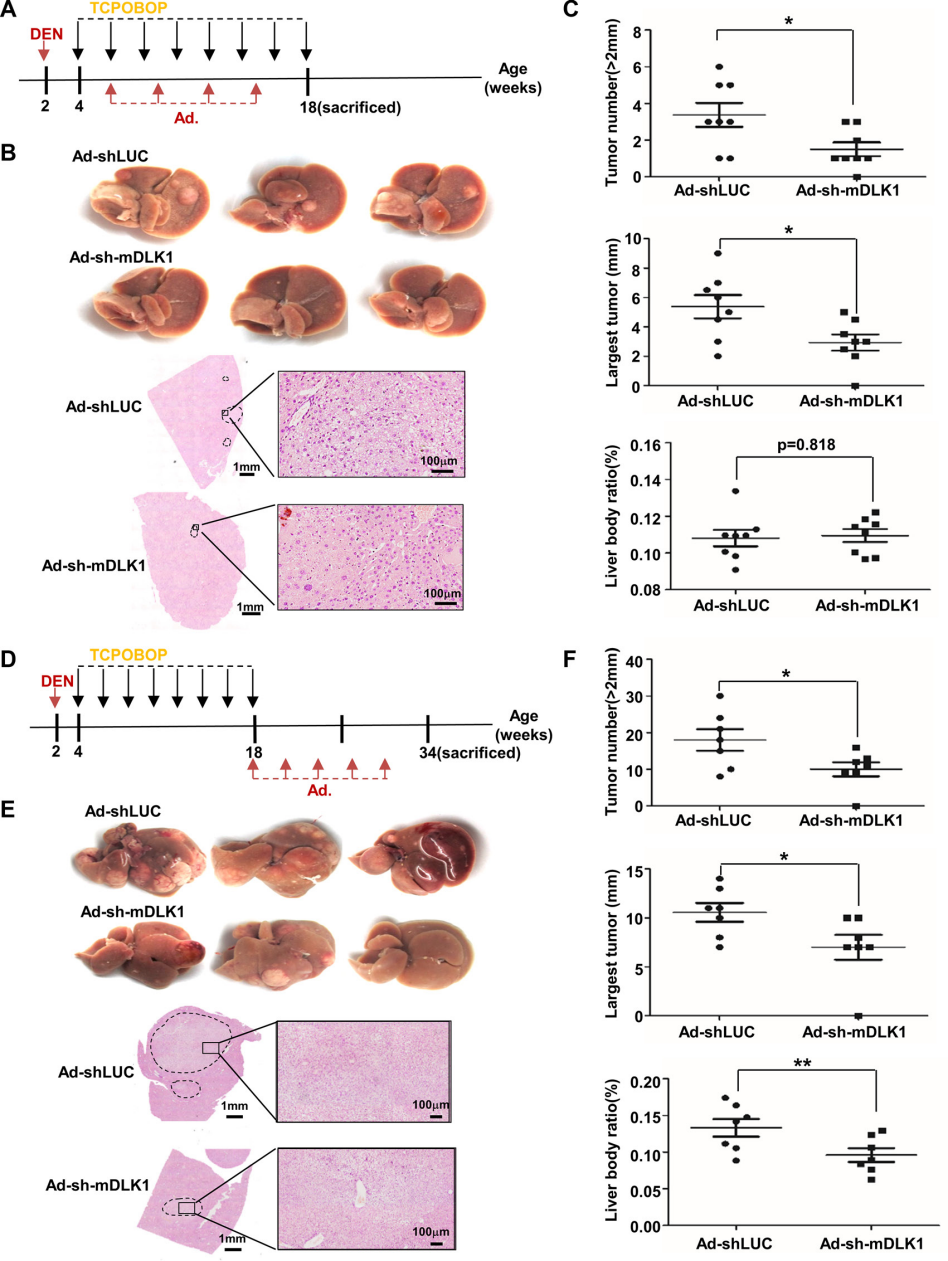
Adenovirus-mediated mouse Dlk1 knockdown attenuates HCC progression (**A**–**C**) The effect of early adenovirus-mediated DLK1 knockdown on DEN-induced mouse HCC tumors. (A) Scheme of adenovirus-mediated mDLK1 knockdown in DEN-induced mouse HCC model. BL/6 mice were treated with DEN plus eight injections of TCPOBOP, where adenovirus administration for four times in total was performed via tail vein injection at an interval of 3 weeks. (**B**) Representative images of livers and H&E-stained sections from the mice were shown. And (C) numbers of tumors (> 2 mm in diameter), size of largest tumors, and liver:body weight ratio were statistically analyzed. (**D**–**F**) The effect of later adenovirus-mediated *Dlk1* knockdown on DEN-induced mouse HCC tumors. (D) Scheme of adenovirus-mediated mDLK1 knockdown in the mouse HCC model. BL/6 mice were treated with DEN plus eight injections of TCPOBOP and either received *Dlk1* shRNA containing adenovirus or control injection starting at week 18 for a total of five times at an interval of 3 weeks. Mice were sacrificed at week 34. (E) Representative images of livers and H&E-stained sections from the mice were shown. (F) Numbers of tumors, size of largest tumors and liver:body weight ratio were statistically analyzed. Data are represented as means ± SD. *p < 0.05; **p < 0.01.

We then evaluated the therapeutic efficacy of the recombinant adenovirus-mediated *Dlk1* knockdown in this model. Here we classified these mice into two groups. The *Early Group* was administrated at 4 weeks after DEN treatment, all eight mice injected with *luciferase* shRNA vector as controls developed tumor nodules in livers when sacrificed at 18 weeks, whereas the other eight mice injected with adenovirus for *Dlk1* knockdown showed the significant reduction of number and size of tumor nodules as compared to the controls (Figure 4A–4C). Endogenous DLK1 knockdown was validated by immunofluorescence assay (Figure S3E). The *Late Group* was administrated at 16 weeks after DEN injection, as expected, seven mice injected with adenovirus for *Dlk1* knockdown showed the significant reduction of tumor number, size and weight, as compared to mice injected with control vector (Figure 4D–4F). These data demonstrated that adenovirus-mediated DLK1 knockdown can suppress tumor progression in the DEN-induced mouse HCC model.

### DLK1 knockdown delays G1-S cell cycle phase transition

**Figure 5:**
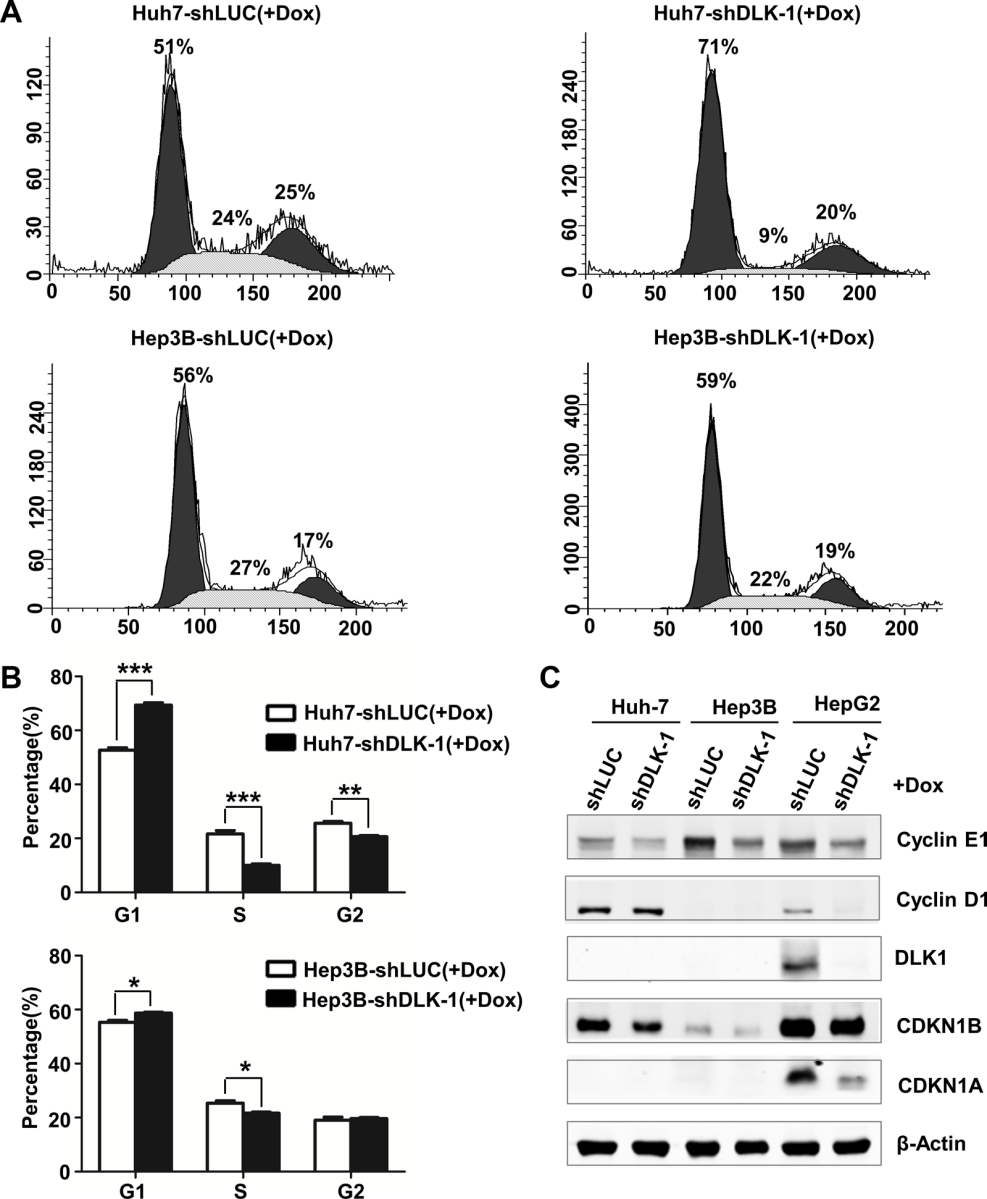
DLK1 downregulation induces an S phase arrest in human HCC cells (**A**) Cell-cycle profiles of lentiviral infected Huh-7 and Hep3B stable cells. Numbers over each histogram indicate the percentage of cells in different phases. (**B**) Statistic analysis of the cell cycle distributions. The columns represent the average value of three independent experiments with standard deviations. *p < 0.05; **p < 0.01. (**C**) Western blot analysis for documenting protein expression levels of cell cycle regulators.

To determine the mechanism by which DLK1 knockdown exerts antitumor activity in HCC cells, the cell cycle progression was analyzed using flow cytometry. Interestingly, cell populations in S phase of Huh-7 and Hep3B cells containing *DLK1* shRNA were significantly reduced, whereas the cell populations in G1 phase were significantly increased, as compared to the control cells respectively (Figure 5A and 5B). It is suggested that DLK1 knockdown may delay G1-S cell cycle phase transition.

Furthermore, we evaluated the key regulators in G1 to S phase transition. As was expected, cyclin E1 level was obviously decreased in both Huh-7 and Hep3B cells as DLK1 was knocked down (Figure 5C and Figure S4). We also evaluated the HepG2 cells with DLK1 knockdown. In addition to cyclin E1, cyclin D1 was also significantly decreased. These data indicated that DLK1 knockdown leads to G1 phase arrest through downregulating certain cell cycle regulators such as cyclins E1 and D1.

### DLK1 knockdown initiates cell differentiation of HCC

**Figure 6:**
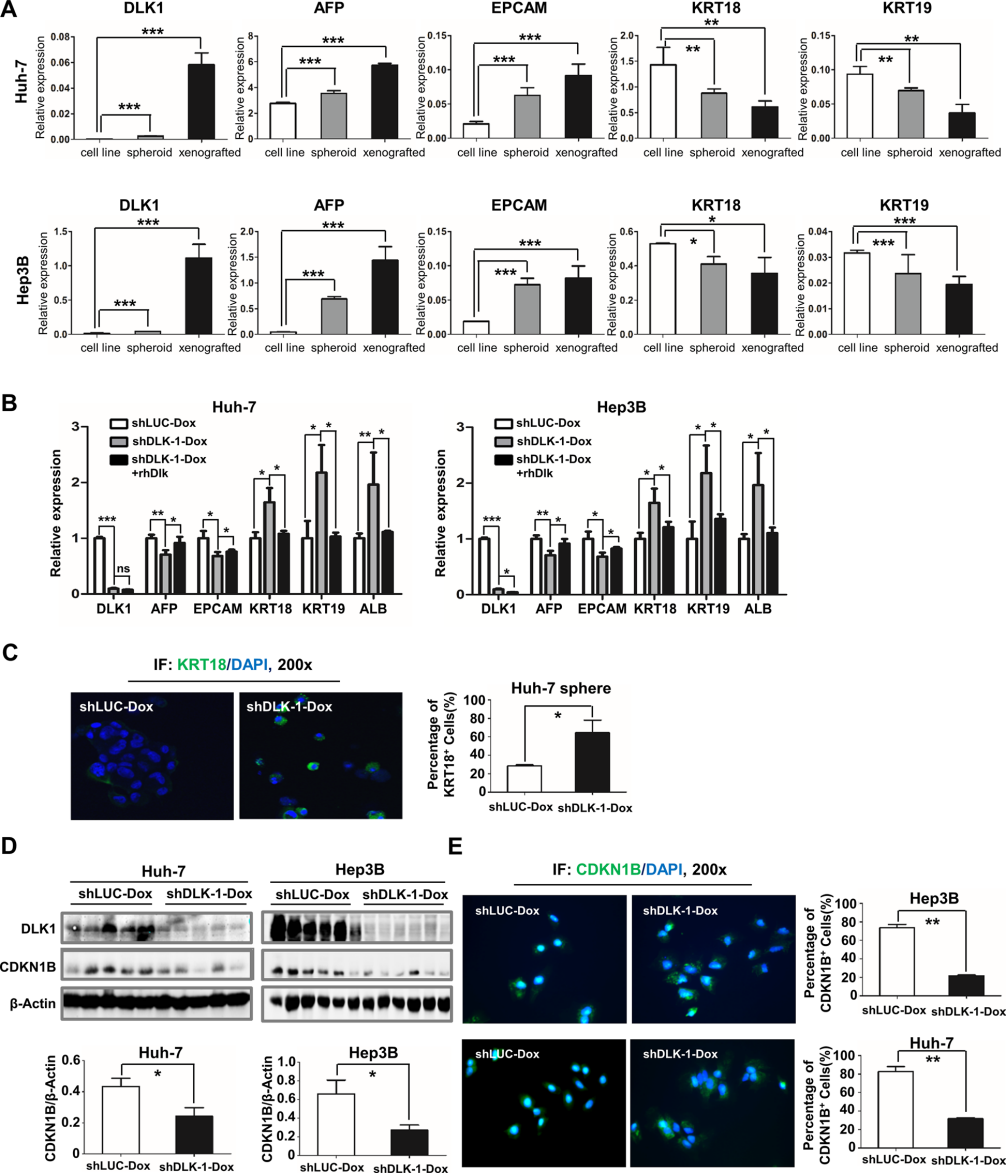
Inducible downregulation of DLK1 promotes cell differentiation of HCC (**A**) Molecular markers for hepatic progenitor and differentiated cells were enriched in spheroid colonies and xenograft tumors. The qRT-PCR data were analyzed by one-way ANOVA. For comparison with expression level of cell line, Student's *t*-test was performed. (**B**) Decreased *AFP* and *EPCAM* as well as increased *KRT18*, *KRT19* and *ALB* in spheroids were detected by quantitative RT-PCR when *DLK1* was knocked down by DOX addition. The expression level of CSC markers was reversed in the regenerated spheroids as recombinant DLK1 protein was introduced to culture medium. The qRT-PCR data were also analyzed by one-way ANOVA, followed by pair-wise comparison. (**C**) Cell spheres were collected for immunofluorescent staining. KRT18^+^ cells were counted from three random fields (mean ± SD) (right). (**D**) Xenograft tumors derived from Huh-7 and Hep3B cells were examined using western blotting assay with anti-CDKN1B antibody (upper) and then CDKN1B level was normalized based on b-actin and statistically analyzed (bottom). (**E**) The immunofluorescence assays depicted the CDKN1B staining (green) and the cell nucleus dyed with DAPI (blue). The right histograms represent the percentage of CDKN1B^+^ cells. *p < 0.05; **p < 0.01; ***p < 0.001.

In this study, we noted that DLK1 expression was dramatically elevated in xenograft tumors (Figure S2C and Figure 3D). It is implied that DLK1^+^ cells, which may represent CSCs, were enriched during *in vivo* tumorigenicity. Here we also examined other molecular markers for hepatic progenitor cells or differentiated cells, including AFP, EPCAM, KRT18 and KRT19. The data indicated that both *AFP* and *EPCAM* were significantly increased in spheroids and xenograft tumors, whereas *KRT18* and *KRT19* were significantly decreased in these samples, as shown in Figure 6A, supporting that CSCs were enriched in spheroids and xenograft tumors.

To explore mechanism of antitumor activity, we examined these xenograft tumors and spheroids with *DLK1* knockdown. Interestingly, both AFP and EPCAM were significantly decreased, and by contrast, KRT18 and KRT19 were significantly increased as compared to the controls (Figure 6B and Figure S5). Immunofluorescent assay also confirmed that KRT18 was significantly elevated in these spheroids derived from Huh-7 cells with *DLK1* knockdown (Figure 6C). These data suggested that the targeting DLK1 may exert antitumor effect via promoting cell differentiation of HCC CSCs.

Cell differentiation is closely associated with cell cycle progression. We also examined some key regulators involved in cell cycle. In addition to cyclins E1 and D1, CDKN1B was decreased as DLK1 was knocked down (Figures 5C and 6D; Figure S4). The decreased nuclear CDKN1B expression was further confirmed by immunofluorescent assay (Figure 6E). Another cdk inhibitor CDKN1A was also decreased in HepG2 cells with DLK1 knockdown (Figure 5C and Figure S4). These data suggested that DLK1 promotes HCC cell differentiation possibly through regulating cell cycle regulators, including active and negative regulators.

It should be pointed out that DLK1 has been reported as a negative regulator of Notch1 activation through interacting with specific EGF-like repeats [24], and also stimulates integrin downstream signaling to activate MEK/ERK and the subsequent upregulation of *Sox9* expression to inhibit adipocyte differentiation [25]. However, here the protein level of NICD and mRNA expression of *SOX9* had no obvious change in HCC cells, as *DLK1* was knocked down (Figures S6A and S6B).

## DISCUSSION

*DLK1* is located within an imprinted *DLK1-DIO3* region of human chromosome 14q32 and widely expressed from the paternal allele in the embryo [7, 8]. However, its expression level was restricted to few tissues and glands after birth [9–11]. Many studies have demonstrated that DLK1 expression is elevated in a variety of tumors due to epigenetic defects [13, 14, 17]. Our previous study also indicated that DLK1 was overexpressed in 73.2% human HCC specimens because of the decreased DNA methylation on the promoter of DLK1 [19]. A recent study demonstrated serum DLK1 is a potential prognostic biomarker in HCC patients [26].

DLK1 as a transmembrane protein is highly expressed in mouse fetal liver. DLK1^+^ cells derived from mouse E14.5 fetal liver showed properties of bipotent progenitor cells [27, 28]. Our previous studies indicated that DLK1 is a molecular biomarker for HCC CSCs, and candidate therapeutic target for HCC treatment [20]. However, the therapeutic efficacy needs to be further validated through *in vivo* experiments. In this study, we employed human HCC xenograft tumor models and the DEN-induced mouse HCC model to address the therapeutic efficacy of targeting endogenous human or mouse DLK1 in HCC cells. In the human HCC xenograft tumor models, including subcutaneous and orthotopic tumor xenografts, *DLK1* knockdown can significantly suppress tumor growth (Figures 2A–2C and 3A–3D; Figure S2A–S2C). In the DEN-induced mouse HCC model, the adenoviral-mediated *Dlk1* knockdown also reduces tumor growth of mouse HCCs (Figures 4A–4C and 4D–4F). These collective data documented that DLK1 is indeed served as a therapeutic target for HCC treatment.

We proposed that the targeting endogenous DLK1 could suppress the malignant behaviors of HCC cells possibly through interfering cancer stem/progenitor cells. Here our experiments showed that DLK1 knockdown could delay the G1 to S phase transition of cell cycle progression, leading to G1 arrest without obvious cell apoptosis (Figures 5A and 5B; Figure S7). Interestingly, the *DLK1* knockdown resulted in the decrease of *AFP* and *EPCAM*, the molecular markers for hepatic progenitor cells, but the increase of *KRT18*, *KRT19* and *ALB*, the markers for the differentiated hepatocytes (Figures 6B and 6C; Figure S5). In previous investigation, CDKN1B has been shown to play an important role in stem and progenitor cells, as characterized in murine hematopoietic system [29, 30]. In these studies, not only CDKN1B increased the proliferation and pool size of hematopoietic progenitor cells, it also cooperated with CDKN1C to maintain hematopoietic stem cell quiescence. In mouse mammary gland, CDKN1B deficiency produced low proliferation, decreased ductal branching and impaired lobuloalveolar differentiation [31]. In this study, CDKN1B was significantly reduced in the xenograft tumors as DLK1 was knocked down (Figure 6D), implying that DLK1 knockdown may initiate cell differentiation of HCC CSCs possibly through regulating cell cycle regulators such as CDKN1B.

In general, our results suggested that targeting DLK1 might inhibit the tumor growth via initiating cell differentiation of HCC CSCs, although molecular mechanisms by which DLK1 knockdown contributes differentiation therapy of HCC should be further investigated.

## MATERIALS AND METHODS

### Cell lines

Human HCC cell lines Huh-7, Hep3B and hepatoblastoma cell line HepG2were purchased from the Institute of Biochemistry and Cell Biology, Shanghai Institutes for Biological Sciences, Chinese Academy of Sciences. Huh-7 and Hep3B cell lines were re-authenticated with DNA (STR) profiling and used within 6 months.

### Animal preparations

Male athymic nude mice and C57BL/6 mice were from Shanghai Laboratory Animal Center, Chinese Academy of Sciences, and allowed to acclimate in the animal facility of Shanghai Research Center for Biomodel Organism with *ad libitum* access to food and water for at least 7 days prior to manipulation. Animals were handled in accordance with the guidelines of the US National Institutes of Health, and all the experimental protocols were approved by the Institutional Animal Care and Use Committee.

### Antibodies and qPCR primers

Mouse monoclonal antibodies against human DLK1 (clone 29A7D11C8B7) were generated by the animal immunization for hybridoma development (ChemPartner, Shanghai, China). The preparation and purification of DLK1 monoclonal antibody were according to the company's procedures. Other antibodies used in this study were described in Supplemental Experimental Procedures. All quantitative RT-PCR primers used in this study can be found in Supplementary Materials.

### Construction of recombinant vectors

Plasmid Tet-pLKO-neo (Addgene plasmid #21916) was used to construct recombinant vector generating small hairpin RNA (shRNA) against human *DLK1*, the stuffer DNA was removed from plasmid Tet-pLKO-neo by AgeI/EcoRI enzyme digest and then replaced with double-stranded oligonucleotides encoding the desired shRNA and AgeI/EcoRI sites. To construct the adenoviral vector containing shRNA against mouse *Dlk1* or control sequence, the plasmid pGCsi-H1/Neo/GFP which was derived from pSuper (Oligoengine, USA) with a recognition site for the restriction endonuclease Pme I destroyed by site-directed mutagenesis was applied. Briefly, the synthesized oligonucleotides encoding the desired shRNA were inserted into pGCsi-H1/Neo/GFP. This plasmid containing shRNA sequence was then digested by Sal I, and the large fragment of gel-extract product was ligated to pShuttle (Stratagene, USA) which was linearized by the same enzyme digestion. The constructed pShuttle-H1-shRNA/GFP was then transformed into *E.coli* BJ5183 to produce recombined plasmid by homologous recombination with pAdEasy-1 (Stratagene, USA). The newly recombined plasmid for adenovirus production was verified by restriction endonuclease digestions. To generate stable luciferase-expressed Huh-7 cell line, the pCDH-*luciferase* plasmid was applied. The ORF (Open Reading Frame) of *luciferase* transcript was amplified from pGL3 plasmid (Promega, USA) and was cloned into the pCDH-CMV-MCS-EF1-copGFP plasmid (System Biosciences, USA). The primers for PCR amplification were as follows: *luciferase* forward primer, 5′- CAGGAATTCATGGAAGACGCCAAAAACAT-3′; *luciferase* reverse primer, 5′- ATAGGATCCTTACACGG CGATCTTTCCGC-3′. All shRNA sequences and the methods of stable cell lines generation were described in Supplementary Experimental Procedures.

### Xenograft tumor model

Inducible stable cells (5 × 10^6^ cells/100 ml) were injected hypodermically into the flanks of 6-week-old male nude mice (BALB/c-*nu/nu*). Dox contained in drinking water at a concentration of 1 mg/ml was given to mice once tumors reached a volume of 150–200 mm^3^. Tumor volume was calculated as width^2^ × length/2 in an interval of 7 days, and animals were sacrificed at 15 days after Dox induced.

### Diethylnitrosamine (DEN)-induced HCC model and administration of the recombinant adenoviral vectors

A two-step strategy was used for hepatocarcinogenesis [23]. Briefly, 14–15 day old mice were injected with DEN (20 mg/kg body weight) (Sigma) to initiate tumor growth. Beginning at day 28, mice were injected with a phenobarbital-like inducer TCPOBOP (3 mg/kg body weight) (Sigma) once every two weeks for a total of eight times to promote tumor growth. Subsequently, these mice were randomly assigned into two groups. *Early Group* were treated with either adenovirus for *Dlk1* knockdown or adenovirus for *luciferase* knockdown as control (5 × 10^9^ particle per animal) intravenously every 3 weeks for a total of 4 times, starting 4 weeks after DEN administration, while *Late Group* were injected with that for a total of 5 times, starting 16 weeks after DEN injection. Finally the mice were sacrificed to evaluate the effect of DLK1 knockdown on hepatocarcinogenesis by examining tumor number, size and weight.

### Orthotopic xenograft mouse model and *in vivo* bioluminescent imaging

For an orthotopic tumor model, approximately 2 × 10^6^ human Huh-7 cells with stable luciferase expression were injected into the left liver lobe of the nude mice in 30 μl of culture medium according to the described previously [32]. For tracing those luciferase-expressing HCC cells in mice, imaging was performed with lumazone imaging system (Mag Biosystems, Tucson, AZ, USA). Here the mice were given by intraperitoneal injections with 200 ml of 15 mg/mL D-luciferin 15 min before imaging. Imaging was performed once every week, and then tumor luminescence in the region of interest was determined using LivingImage software and expressed as photons/sec. The mice with an orthotopic tumor without statistic difference in luminescence were separated into two groups, and then injected with the recombinant adenoviruses for endogenous *Dlk1* knockdown and control adenovirus, respectively. After 5 weeks of adenovirus administration, the mice were sacrificed and examined.

### Statistical analyses

Statistical analysis was conducted by Student's *t*-test for two groups. Comparison among more than 3 groups was done using one-way ANOVA (Prism Software, Graph Pad Software, Inc.). Data presented in figures were derived from several independent replicates and were showed as mean ± SD. *p* < 0.05 which was considered statistically significant.

## SUPPLEMENTARY MATERIALS FIGURES


