# Supplementary material for: Targeting endogenous DLK1 exerts antitumor effect on hepatocellular carcinoma through initiating cell differentiation
Source: Oncotarget. 2016 Sep 23;7(44):71466–76. doi: 10.18632/oncotarget.12214 (PMC5342093; doi:10.18632/oncotarget.12214)
Supplement: Supplementary file 1 [file oncotarget-07-71466-s001.pdf]

Targeting endogenous DLK1 exerts antitumor effect on hepatocellular carcinoma through initiating cell differentiation

Supplementary Materials

SUPPLEMENTARY EXPERIMENTAL PROCEDURES

Cell culture

AD293, HEK293T, and Human HCC cell lines PLC/PRF/5, Sk-hep1, YY-8103, SMMC7721, HepG2, Hep3B, Huh-7, MHCC-97H, MHCC-97L, MHCC-LM3, MHCC-LM6 and human normal liver cell line L02 were cultured in high glucose (4.5 g/l) DMEM supplemented with 10% fetal bovine serum (FBS), penicillin, and streptomycin. HepG2 cells were cultured in EMEM supplemented with 10% FBS, penicillin, and streptomycin.

RNA isolation and qRT-PCR

Total RNA isolation, cDNA synthesis and quantitative real-time q-PCR were performed as previously published [1]. Quantitative real-time reverse transcription (qRT)-PCR was conducted with the primers listed in Supplementary Table S1. Each sample was analyzed in triplicate. The data was normalized to GAPDH before comparative analysis.

Western blotting analysis

Antibodies for immunoblotting were as follows: anti-DLK1 mouse monoclonal (1:1000, clone:29A7D11C8B7, made in laboratory), anti-GAPDH rabbit monoclonal (1:1000, #5174, CST), anti-NICD polyclonal (1:500, ab8925, Abcam), anti-β-actin mouse monoclonal (1:1000, sc-47778, Santa Cruz), anti-CDKN1B rabbit polyclonal (1:200, sc-528, Santa Cruz), anti-CDKN1A mouse monoclonal (1:200, sc-53870), anti-cyclin E1 mouse polyclonal (1:200, sc-247), anti-cyclin D1 mouse polyclonal (1:200, sc-8396), IRDye®800CW goat anti-rabbit IgG (1:1000, 611-145-002, Rockland), and IRDye®800CW goat anti-mouse IgG (1:1000, 610-145-002, Rockland). Western blot analysis was performed according to the experimental procedure described in earlier study [2].

Lentiviral production and generation of stable cell lines

The lentiviral vectors Tet-pLKO-neo and pLKO.1-TRC, for construction of short hairpin RNA (shRNA) were kindly provided by Dr. Dmitri Wiederschain (Addgene plasmid #21916) and Dr. David Root (Addgene plasmid #10878). Lentiviruses were generated by co-transfecting 293T cells with 4 μg of shRNA-encoding plasmid and 3 μg of pLp1, 3μg pLp2 and 2 μg of pLp/VSVG plasmids using lipofectamine2000 (Invitrogen) in each 100 mm petri dish. Growth media was exchanged the following day and lentivirus-containing supernatant was harvested 48 hours later. Stable cell lines expressing inducible shRNAs were generated by lentiviral infection using the Tet-pLKO-neo lentiviral expression system in the presence of 5 μg/ml polybrene, followed by G418 selection (600–1000 μg/ml). These cell lines were maintained in media with tet-free serum (Clontech Laboratories Inc.). Stable cell lines expressing constitutive shRNAs were generated by lentiviral infection using the pLKO.1-TRC system followed by puromycin selection (1–10 μg/ml). The majority of target-specific shRNA sequences used in this study was obtained from previous studies [3, 4]. The insert sequences for shDLK-1 are 5′-CCGGGGTGTCCATGAAAGAGCTCCTCG AGGAG CTCTTTCATGGACACCTTTTT-3′ (sense strand) and 5′-AATTGGTGTCC ATGAAAGAGCTCCTCGAGGAG CTCTTTCATGGACACC-3′ (antisense strand). The insert sequences for shDLK-2 include 5′-CCGGGATCGACA TGACCACCTTC CTCGAGGAAGGTGGTCATGTCGA TCTTTTT-3′ (sense strand) and 5′-AATTGATCGACAT GACCACCTTCCTCGAGGAAGGTGGTCATGTCGAT C-3′ (antisense strand). The control sequences for shLUC are 5′-CCGGCTTACGCTGAGTACTTCG ACTCGAGTCGAAGTACTCAGCGTAAGTTTTT-3′ (sense strand) and 5′-AATTCT TACGCTGAGTACTTCGA CTCGAGTCGAAGTACTCAGCGTAAG-3′ (antisense strand). The lentiviral vector pCDH-CMV-MCS-EF1-GreenPuro for construction of cDNA was purchased from Systembiosciences, Inc. Lentiviral particles were

Supplementary Table S1: Sequences of designed primers for quantitative real-time PCR analysing

Genes		Sequence (5′-3′)	PCR products
DLK1	Forward	GCACTGTGGGTATCGTCTTCC	113 bp
	Reverse	CTCCCCGCTGTTGTACTGAA	
Mouse Dlk1	Forward	CCCAGGTGAGCTTCGAGTG	216 bp
	Reverse	GGAGAGGGGTACTCTTGTTGAG	
GAPDH	Forward	CTGGGCTACACTGAGCACC	101 bp
	Reverse	AAGTGGTCGTTGAGGGCAATG	
Mouse Gapdh	Forward	TGGCCTTCCGTGTTCCTAC	178 bp
	Reverse	GAGTTGCTGTTGAAGTCGCA	
AFP	Forward	AGTGAGGACAAACTATTGGCCT	98 bp
	Reverse	ACACCAGGGTTTACTGGAGTC	
CK19	Forward	TGAGTGACATGCGAAGCCAAT	98 bp
	Reverse	CTCCCGGTTCAATTCTTCAGTC	
CK18	Forward	GTTGACCGTGGAGGTAGATGC	86 bp
	Reverse	GAGCCAGCTCGTCATATTGGG	
ALB	Forward	TTTATGCCCCGGAACTCCTTT	148 bp
	Reverse	AGTCTCTGTTTGGCAGACGAA	
EPCAM	Forward	ATAACCTGCTCTGAGCGAGTG	99 bp
	Reverse	TGCAGTCCGCAAACTTTTACTA	
SOX9	Forward	AGCGAACGCACATCAAGAC	85 bp
	Reverse	CTGTAGGCGATCTGTTGGGG	

generated by cotransfection using lipofectamine2000 of 293T cells with lentiviral constructs and helping plasmids psPAX2 and pMD2.G which were both gifts from Didier Trono (Addgene plasmid # 12259 and # 12260). Lentiviral particles were harvested and stored. Stable Huh-7 cell line continuously expressing firefly luciferase was generated by viral infection and was followed by puromycin selection (10 μg/ml).

Recombinant adenovirus construction and purification

Recombinant adenoviruses were constructed using the AdEasy™ XL system (#240010; Stratagene, CA, USA) following the manufacturer's instructions. A sequence (5′-AATGGAGTCTGCAAGGAAC-3′) corresponding to nucleotides 346–364 of mouse *DLK1* mRNA [5] (GenBank accession number NM_010052.2) was designed as short hairpin oligonucleotides to insert into pShuttle plasmid which was modified by cloning DNA fragments encoding GFP protein, under the transcriptional control of the active human H1 promoter. Adenoviruses were generated by transfecting linearized recombined plasmid DNA into AD293 cells using Lipofectamine2000. Viruses were amplified and purified by cesium chloride density-gradient centrifugation. Virus titer was determined by plaque assay. The other target sequences used for adenovirus generation in this study were described previously and listed below:

Target sequence for shDLK-1: 5′-GGTGTCCATGA AAGAGCTC-3′

Target sequence for shNC (scramble): 5′-AGCGTT CACTCCCAACCTG-3′

Target sequence for shLUC: 5′-CTTACGCTG AGTACTTCGA-3′

Colony formation assay

Inducible shRNA-expression stable cells were plated at 1×10^3 cells per well in a six-well plate. Cells were cultured with DMEN containing 10 ng/ml doxycycline. Three weeks later, cells were fixed in methanol, stained with 0.1% (w/v) crystal violet, and washed with water. The cell colonies were photographed and the number of colonies > 100 mm in diameter were counted using imageJ software.

Sphere formation assay

Inducible stable cells were seeded at a density of 10 cells/ul on 6-well ultra-low adherent plates containing 2 ml basal culture medium(DMEM-F12,1:1; Invitrogen, San Diego, CA) with 1XGlutamine, B27 (Invitrogen-Gibco, Grand Island, NY), 100 U/mL penicillin and

100 g/mL streptomycin. Culturing was conducted in a humidified incubator in an atmosphere of 5% CO_2, 20 ng/mL bFGF, and 20 ng/mL EGF were added to the medium every other day. After two weeks, only cell clusters with a diameter of > 50 µm were counted as primary spheres.

Cell cycle and cell apoptosis analysis

Lentivirus infected Hep3B and Huh-7 cells were plated after selection; 24 hours later, cells were harvested and fixed in 70% ethanol, resuspended in PBS, and treated with RNase A (10 mg/mL) and propidium iodide (10 µg/mL) for 30 min each. Samples were measured using a FACSCalibur flow cytometer, CellQuest (BD Biosciences, USA). Apoptotic cells were detected by Annexin V-FITC apoptosis detection kit (BMS500FI, eBioscience) according to the protocol recommended by the manufacturer.

Hematoxylin and eosin staining

Liver tumor tissues were fixed in 4% buffered paraformaldehyde and subsequently treated for the histological study by dehydration (increasing alcohol concentrations, from 60% to absolute alcohol), mounting in xylene, and immersion in paraffin. The paraffin blocks were cut into 4 mm sections for hematoxylin-eosin (H&E) staining.

Immunofluorescence assay

An immunofluorescence assay was used to detect CDKN1B expression in HCC cell lines following standard procedures. In brief, cells which were grown on coverslips were fixed in 4% paraformaldehyde for 15 min at room temperature (RT). Aspirate fixative, rinse in 1X PBS and followed by specimen blocking. After blocking for 1 hour at RT in blocking buffer (PBS containing 5% donkey serum and 0.3% Triton X-100), specimen were incubated overnight at 4°C with anti-CDKN1B antibody (1:200) in blocking buffer. Following three PBST washes, specimen were incubated with the AlexaFluor 488-Donkey anti-Rabbit IgG (1:500, Molecular Probe, USA) at 37°C for 1 hour. Rinse again and coverslip slides with mounting solution (Sigma, USA) with DAPI (1000 ng/ml, Vysis Inc). The slides were observed under a fluorescence microscope (BX-60; Olympus) and an image analyzer system. For KRT18 detection of HCC spheroid colonies, the cell spheres were incubated with anti-KRT18 antibodies (1:200), with Alexa Fluor 488-coupled secondary antibodies (1:500, Molecular probe). The stained cells were observed by Zeiss confocal microscopy and a ZEISS LSM Image Browser (Carl Zeiss, Germany).

Immunohistochemistry staining

Tissues were collected in 4% paraformaldehyde (PFA), embedded in paraffin following standard procedures. For CK18 detection, slides were brought to a boil in 10 mM sodium citrate buffer (pH 6.0) for antigen retrieval and then incubated in 3% hydrogen peroxide to remove the endogenous peroxidase activity. After blocking for 1 hour at RT in blocking buffer (PBS containing 10% goat serum and 0.2% Triton X-100), slides were incubated overnight at 4°C with anti-KRT18 antibody (1:200, #4548, CST), anti-KRT19 antibody (GT225502, Gene Tech, Shanghai), anti-AFP antibody (GA000802, Gene Tech, Shanghai) and anti-EPCAM antibody (1:100, #14452, CST) diluted in blocking buffer. Following three PBST washes, slides were incubated with the horseradish peroxidase (HRP)-conjugated antibodies (DACO, Kyoto, Japan) at 37°C for 1 hour. The signals were detected by Diaminobenzidine (DAB) Substrate Kit (Vector Laboratories, Inc., Burlingame, CA) according to the manufacturer's instructions.

REFERENCES

1. Huang J, Zhang X, Zhang M, Zhu JD, Zhang YL, Lin Y, Wang KS, Qi XF, Zhang Q, Liu GZ, Yu J, Cui Y, Yang PY, et al. Up-regulation of DLK1 as an imprinted gene could contribute to human hepatocellular carcinoma. Carcinogenesis. 2007; 28:1094–103.
2. Wang YP, Huang LY, Sun WM, Zhang ZZ, Fang JZ, Wei BF, Wu BH, Han ZG. Insulin receptor tyrosine kinase substrate activates EGFR/ERK signalling pathway and promotes cell proliferation of hepatocellular carcinoma. Cancer Lett. 2013; 337:96–106.
3. Kim Y, Lin Q, Zelterman D, Yun Z. Hypoxia-regulated delta-like 1 homologue enhances cancer cell stemness and tumorigenicity. Cancer Res. 2009; 69:9271–80.
4. Xu X, Liu RF, Zhang X, Huang LY, Chen F, Fei QL, Han ZG. DLK1 as a potential target against cancer stem/progenitor cells of hepatocellular carcinoma. Mol Cancer Ther. 2012; 11:629–38.
5. Bauer M, Szulc J, Meyer M, Jensen CH, Terki TA, Meixner A, Kinkl N, Gasser T, Aebischer P, Ueffing M. Delta-like 1 participates in the specification of ventral midbrain progenitor derived dopaminergic neurons. J Neurochem. 2008; 104:1101–15.

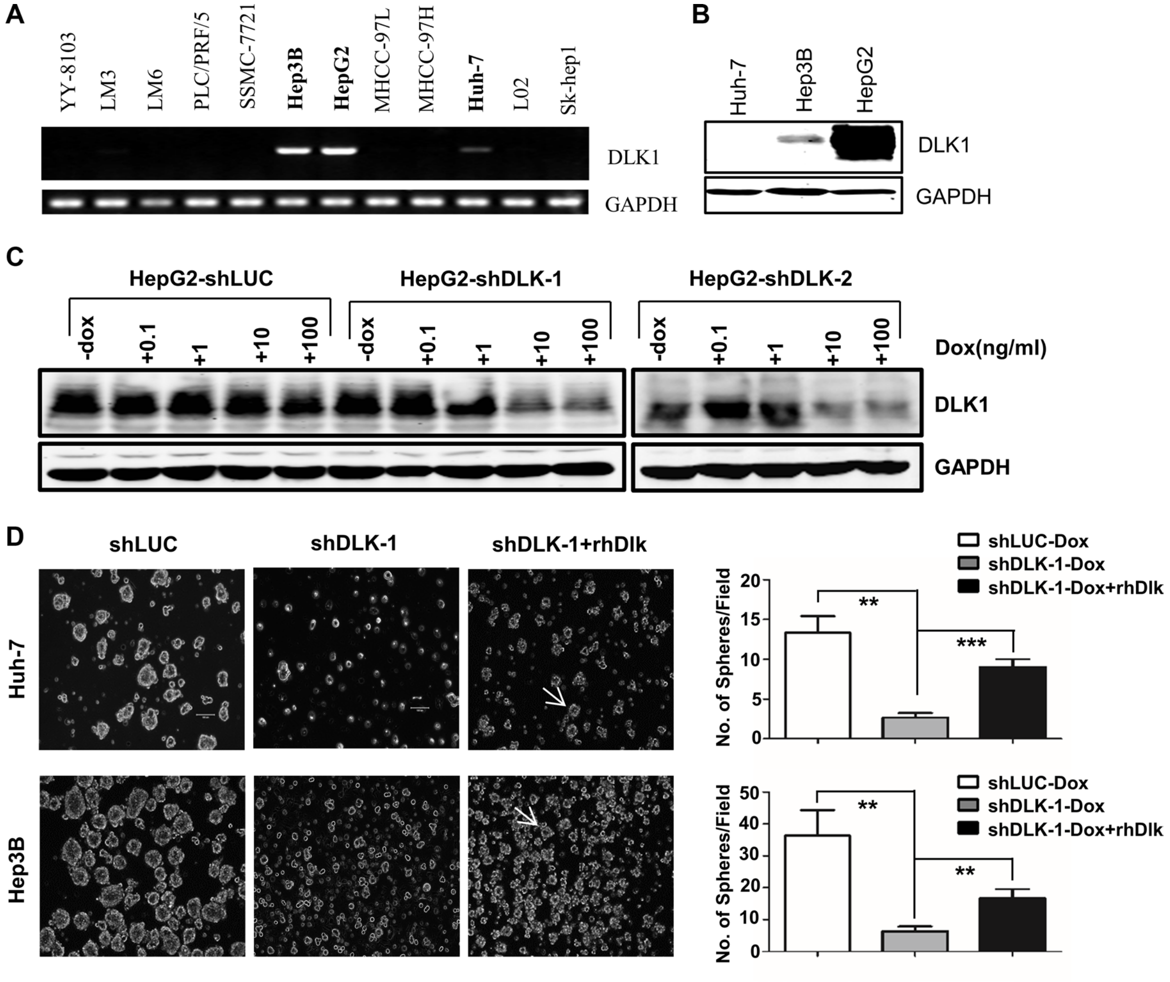

Supplementary Figure S1: Inducible knockdown of *DLK1* and the rescue assay of spheroid formation in HCC. (**A**) *DLK1* mRNA was evaluated by qRT-PCR in a panel of HCC cell lines. (**B**) *DLK1* protein was evaluated by Western blot assay in Huh-7, Hep3B and HepG2 cells. (**C**) DLK1 knockdown was induced in HepG2 cells with stably expressed plasmid encoding shRNA by Dox in a dose-dependent manner. Here Western blotting assay was employed to detect DLK1 level at three days after Dox treatment, where HepG2 cells with *luciferase* shRNA vector were used as negative control. (**D**) DLK1 reintroducing could rescue the spheroid formation of *DLK1*-silenced HCC cells. Huh-7 and Hep3B stable cells expressing *DLK1* shRNA were treated by adding recombinant DLK1 protein (rhDlk, Enzo lifescience) at a reference concentration of 50 ng/ml into the culture medium. The number of spheroids were counted after 7 days. Data were analyzed by one-way ANOVA($p < 0.001$ for both cell lines), followed by pairwise comparison (**$p < 0.01$ versus their respective shLUC-Dox, ***$p < 0.001$ versus shDLK-1-Dox in Huh-7 cells and **$p < 0.01$ in Hep3B cells).

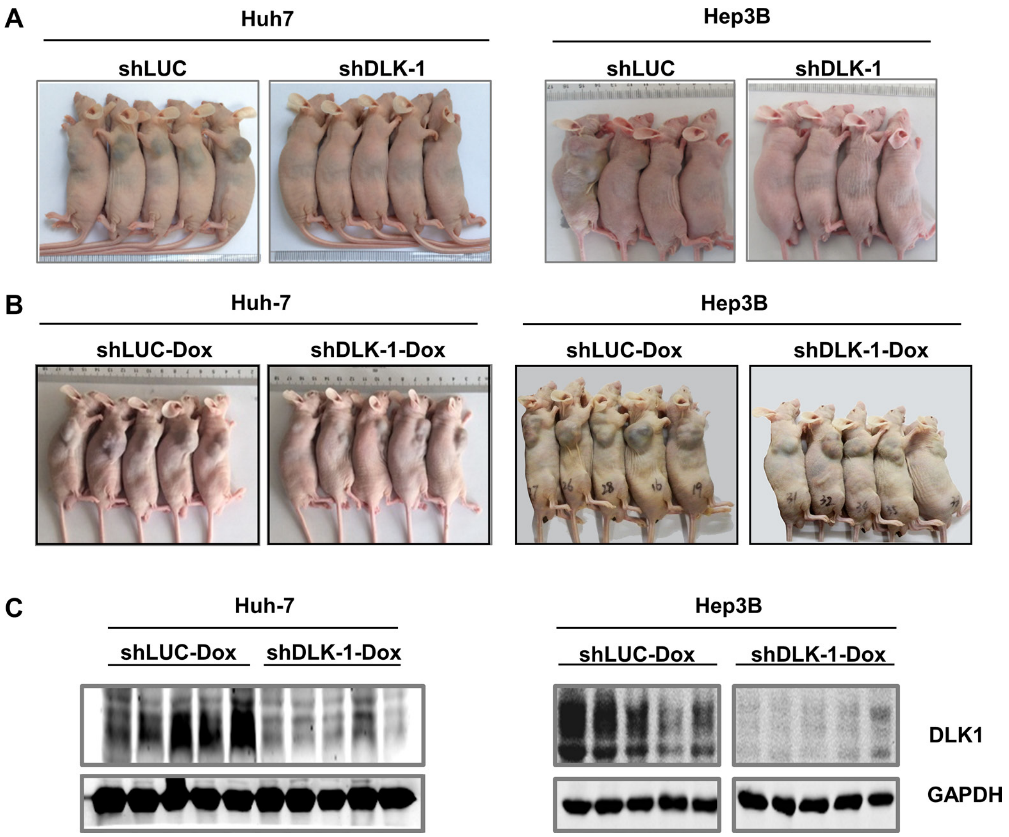

Supplementary Figure S2: Downregulation of DLK1 inhibits the xenograft tumor growth by using constitutive and inducible shRNA system. (**A**) Representative images of xenografted mice subcutaneously inoculated by injection of Huh-7 and Hep3B cells with constitutive DLK1 knockdown and controls. (**B**) Representative images of xenografted mice with the inducible DLK1 knockdown, where nude mice bearing xenograft tumors of 150–200 mm^3 were administered 1 mg/ml of Dox for 15 days. (**C**) Western blot analysis was used for evaluating the efficiency of the inducible DLK1 knockdown in these xenograft tumors.

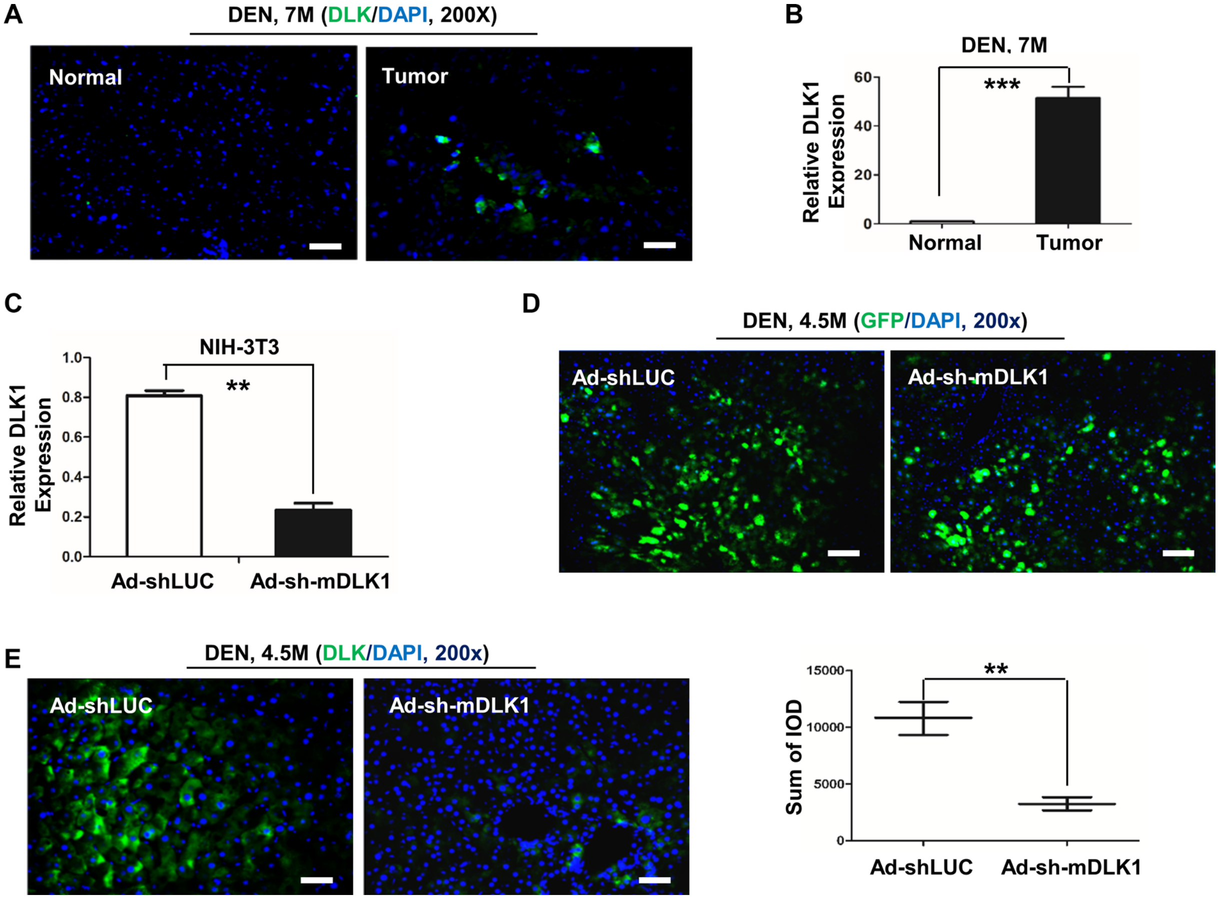

Supplementary Figure S3: Downregulation of DLK1 by transduction of adenovirus containing shRNA cassette *in vitro* and *in vivo*. DLK1 level was evaluated in mouse liver tumors induced by DEN for 7 months using immunofluorescence assay (**A**) Bar = 100 µm) and qRT-PCR (**B**) where adjacent non-tumor liver was used as control. (**C**) qRT-PCR showing mouse *Dlk1* expression in NIH-3T3 cells transiently transfected with the indicated adenoviruses. (**D**) Fluorescent microscopy showing efficient transduction of hepatocytes, as indicated by GFP expression, 10 days following adenovirus administration. (**E**) The efficiency of adenovirus-mediated mouse DLK1 knockdown was assessed in liver tumors at 21 days following adenovirus administration by immunofluorescence assay. The representative images were shown (left) and the sum of integrated optical density was statistically analyzed (right).

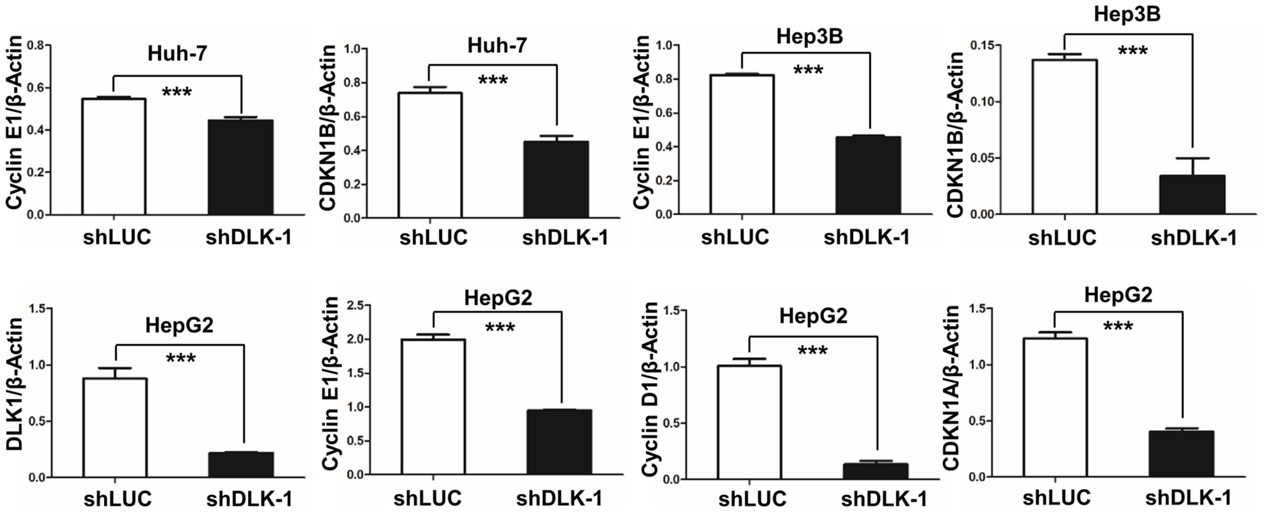

Supplementary Figure S4: The alterations of cell cycle regulators as DLK1 knockdown. The relative quantification of bands in Figure 5C was performed by the optical density scanning by using ImageJ software. The protein level of cyclin E1, cyclin D1, CDKN1B and CDKN1A was downregulated as DLK1 was knocked down in HCC cells. ****$p < 0.001$.

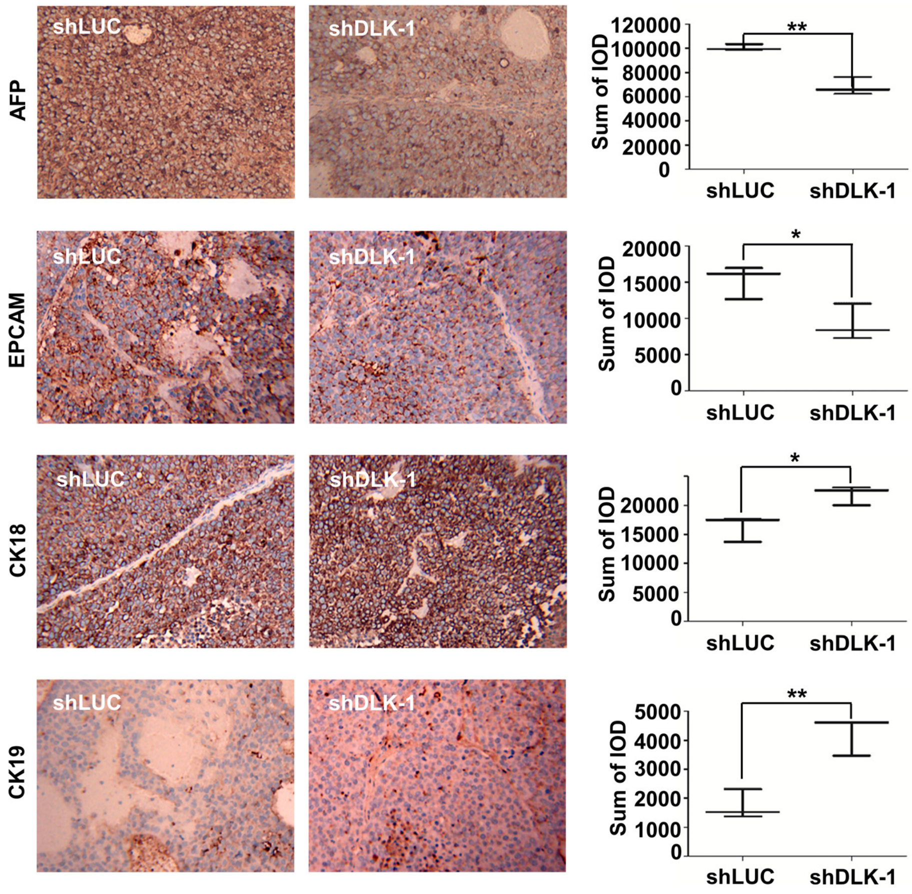

Supplementary Figure S5: Immunohistochemistry staining analysis of CSC markers in orthotopic xenograft tumors. By immunohistochemistry staining, the expression level of both CSC markers AFP and EPCAM decreased in xenograft tumors derived from Huh-7 stable cells in which DLK1 was knocked down as compared to the control cells, while the change of biomarkers KRT18 and KRT19 was opposite.(*$p < 0.05$, **$p < 0.01$, *t* test).

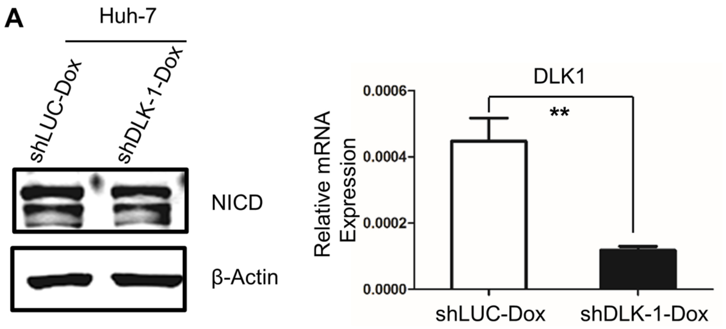

Supplementary Figure S6: DLK1 knockdown does not change the activity of Notch signaling and mRNA levels of *SOX9* in HCC cells. (**A**) Western blot analysis showed the expression of Notch intracellular domain (NICD) in Huh-7 cells with DLK1 knockdown. The efficacy of *DLK1* knockdown was evaluated by qRT-PCR (right). (**B**) *SOX9* mRNA level was analyzed by qRT-PCR (**$p < 0.01$, *t* test).

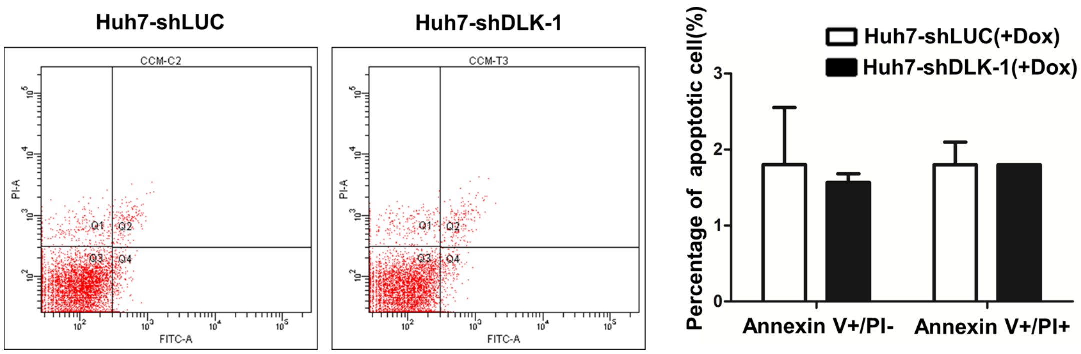

Supplementary Figure S7: DLK1 knockdown does not change the level of cell death in HCC cells. Annexin V positive and Annexin V/PI double positive cells were isolated from Huh-7 stable cells expressing *DLK1* or *luciferase* shRNA and counted by FACs. The percentage of apoptotic cell present in the right histogram.
